# Supplementary material for: A Comprehensive Assessment of Ultraviolet-Radiation-Induced Mutations in Flammulina filiformis Using Whole-Genome Resequencing
Source: J Fungi (Basel). 2024 Mar 20;10(3):228. doi: 10.3390/jof10030228 (PMC10971301; doi:10.3390/jof10030228)
Supplement: Supplementary file 1 [file jof-10-00228-s001.zip › Supplementary Material S8/KEGG annotation/out/64381550635650.os/KO/out_map/map04730.html]

KEGG PATHWAY: Long-term depression - Reference pathway


|  |  |
| --- | --- |
| **Long-term depression - Reference pathway** |  |

[
Pathway menu
| Organism menu
| Pathway entry
| Show description
| User data mapping
]

Cerebellar long-term depression (LTD), thought to be a molecular and cellular basis for cerebellar learning, is a process involving a decrease in the synaptic strength between parallel fiber (PF) and Purkinje cells (PCs) induced by the conjunctive activation of PFs and climbing fiber (CF). Multiple signal transduction pathways have been shown to be involved in this process. Activation of PFs terminating on spines in dendritic branchlets leads to glutamate release and activation of both AMPA and mGluRs. Activation of CFs, which make multiple synaptic contacts on proximal dendrites, also via AMPA receptors, opens voltage-gated calcium channels (VGCCs) and causes a generalized influx of calcium. These cellular signals, generated from two different synaptic origins, trigger a cascade of events culminating in a phosphorylation-dependent, long-term reduction in AMPA receptor sensitivity at the PF-PC synapse. This may take place either through receptor internalization and/or through receptor desensitization.

|  |  |
| --- | --- |
| Reference pathway | 100% |
